# Insights into Autophagy in Microbiome Therapeutic Approaches for Drug-Resistant Tuberculosis

**DOI:** 10.3390/cells14070540

**Published:** 2025-04-03

**Authors:** Md Abdur Rahim, Hoonhee Seo, Indrajeet Barman, Mohammed Solayman Hossain, Md Sarower Hossen Shuvo, Ho-Yeon Song

**Affiliations:** 1 Department of Microbiology and Immunology, School of Medicine, Soonchunhyang University, 31, Suncheonhyang 6-gil, Dongnam-gu, Cheonan-si 31151, Republic of Korea; 2 Human Microbiome Medical Research Center (HM·MRC), School of Medicine, Soonchunhyang University, 22, Soonchunhyang-ro, Sinchang-myeon, Asan-si 31538, Republic of Korea; 3 Probiotics Microbiome Commercialization Research Center (PMC), Soonchunhyang University, 22, Soonchunhyang-ro, Sinchang-myeon, Asan-si 31538, Republic of Korea

**Keywords:** drug-resistant tuberculosis, microbiome therapeutics, autophagy

## Abstract

Tuberculosis, primarily caused by *Mycobacterium tuberculosis*, is an airborne lung disease and continues to pose a significant global health threat, resulting in millions of deaths annually. The current treatment for tuberculosis involves a prolonged regimen of antibiotics, which leads to complications such as recurrence, drug resistance, reinfection, and a range of side effects. This scenario underscores the urgent need for novel therapeutic strategies to combat this lethal pathogen. Over the last two decades, microbiome therapeutics have emerged as promising next-generation drug candidates, offering advantages over traditional medications. In 2022, the Food and Drug Administration approved the first microbiome therapeutic for recurrent *Clostridium* infections, and extensive research is underway on microbiome treatments for various challenging diseases, including metabolic disorders and cancer. Research on microbiomes concerning tuberculosis commenced roughly a decade ago, and the scope of this research has broadened considerably over the last five years, with microbiome therapeutics now viewed as viable options for managing drug-resistant tuberculosis. Nevertheless, the understanding of their mechanisms is still in its infancy. Although autophagy has been extensively studied in other diseases, research into its role in tuberculosis is just beginning, with preliminary developments in progress. Against this backdrop, this comprehensive review begins by succinctly outlining tuberculosis’ characteristics and assessing existing treatments’ strengths and weaknesses, followed by a detailed examination of microbiome-based therapeutic approaches for drug-resistant tuberculosis. Additionally, this review focuses on establishing a basic understanding of microbiome treatments for tuberculosis, mainly through the lens of autophagy as a mechanism of action. Ultimately, this review aims to contribute to the foundational comprehension of microbiome-based therapies for tuberculosis, thereby setting the stage for the further advancement of microbiome therapeutics for drug-resistant tuberculosis.

## 1. Introduction

Tuberculosis (TB), caused by *Mycobacterium tuberculosis* (*M. tb*), is still considered a primary global health concern, causing millions of deaths annually [1]. According to the World Health Organization (WHO)’s 2024 report, 1.25 million people, including 1.09 million HIV-negative individuals and 161,000 HIV-positive individuals, succumbed to this lethal pathogen globally in 2023 [2]. Additionally, the report indicated a continuous increase in new TB cases, totaling 10.8 million in 2023, up from 10.7 million in 2022, 10.4 million in 2021, and 10.1 million in 2020. The global TB incidence rate is estimated to have risen by 4.6% during this period. Furthermore, in 2023, 3.2% of new TB cases and 16% of previously treated cases were classified as multidrug-resistant (MDR) [2]. The report also revealed that 79% of people (3.4/4.3 million) diagnosed with bacteriologically confirmed pulmonary TB were tested for rifampicin resistance in 2023, an increase from 73% (2.9/4.0 million) in 2022 and 69% (2.4/3.5 million) in 2021 [2]. Among those tested, 159,684 individuals with MDR/RR-TB and 28,982 with pre-XDR-TB or XDR-TB were identified in 2023, a significant increase from the previous year [2].

The current regimen for effective TB treatment has utilized a combination of four primary drugs since the 1940s: isoniazid, rifampicin, pyrazinamide, and ethambutol [3]. Despite appropriate initiatives, drug-resistant *M. tb* strains have emerged, attributed to incorrect prescription practices and patient non-compliance [3]. Treating these resistant strains necessitates prolonged treatment periods that are costly and insufficiently effective in completely eradicating *M. tb* pathogens from the host [4,5,6]. Concurrently, this treatment induces adverse and toxic side effects, including the disruption of normal microbiota, irreversible damage to lung tissue, and drug interactions, which complicate patient adherence to treatment regimens [7,8,9].

In light of the preceding context, there is a need for novel therapeutics and new management strategies to combat both drug-sensitive and drug-resistant *M. tb* strains. In this regard, microbiome-based therapeutics represent an underexplored approach that could stimulate host defense mechanisms or mitigate tissue damage associated with infections rather than directly targeting pathogenic components [10,11]. In this context, autophagy is emerging as a potential therapeutic target, and its applicability in tuberculosis management has been substantiated by multiple studies, attracting significant research interest [12,13]. Given these circumstances, probiotics, as a component of microbiome therapeutics, may serve as promising drug candidates due to their documented roles as autophagy activators and subsequent health benefits to the host, as demonstrated experimentally [14,15,16]. Nonetheless, probiotics-mediated autophagy and its consequent role in tuberculosis management remain nascent, necessitating further comprehensive studies.

In this review, we provide a concise overview of microbiome therapeutics and examine the role of autophagy as their underlying mechanism in response to TB. We also discuss the potential of probiotics as emerging microbiome therapeutics that can enhance autophagy, address the challenges, and offer future perspectives on using microbiome therapeutics targeting autophagy to fight tuberculosis. Ultimately, we aim to establish a foundational understanding of the interplay among microbiome therapeutics, autophagy, and tuberculosis.

## 2. Human Microbiome and Its Relation in Terms of Health and Disease

The trillions of microbes, comprising bacteria, fungi, archaea, protozoa, and viruses, may exist in symbiotic, commensal, or pathogenic relationships with their hosts and are collectively called the microbiota. These organisms have colonized the human body for millions of years, establishing a mutualistic relationship through co-evolution with their mammalian hosts to maintain health [17]. This mutualistic role of microbes in human health has unveiled substantial potential for therapeutic applications in disease management, termed microbiome therapy. Microbiome therapy holds immense promise for treating severe diseases and provides a route to personalized medicine, addressing challenges such as interpersonal variability and environmental stability [18]. Given its significance in health, extensive projects like the Human Microbiome Project and MetaHIT (the European Union Project on Metagenomics of the Human Intestinal Tract) have been conducted, focusing on microbiome research across various human body organs [19,20]. Meanwhile, the development of large-scale sequencing technologies, including next-generation sequencing (NGS), has facilitated microbiome engineering and the creation of extensive microbiome databases [21]. Consequently, there is a growing interest in developing and utilizing microbiome-based therapeutics to promote health benefits.

Environmental factors such as antibiotic use or dietary changes can disturb the normal microbiota pattern, disrupting the mutualistic relationship between humans and microbes. This disruption leads to reduced bacterial diversity or altered species abundance [22], a condition known as microbial dysbiosis [23], which is strongly associated with numerous metabolic and infectious diseases [22,24,25,26,27,28]. In vivo studies have demonstrated that dysbiosis drives disease progression and that restoring microbiome consortia can mitigate these conditions [29,30]. Evidence indicates that human microbiota dysbiosis can modulate susceptibility to *M. tb* infection and progression from latent tuberculosis infection (LTBI) to active TB [31]. Additionally, metabolites derived from the microbiome are shown to have direct or indirect effects on host metabolism, thus providing beneficial effects [32]. Moreover, they can regulate immune function, thereby contributing to overall well-being [32].

## 3. Autophagy: An Untapped Mechanism of Action in Microbiome Therapeutics

Autophagy, a highly regulated cellular process essential for maintaining cellular homeostasis, degrades and recycles damaged organelles and proteins while playing a crucial role in clearing pathogens from host cells [33]. It is initiated with the formation of a double-membraned structure known as an autophagosome that encloses cytoplasmic targets and subsequently fuses with lysosomes. This fusion results in the formation of autolysosomes, leading to the degradation of enclosed material by lysosomal enzymes [33]. This dynamic process fulfills various physiological and pathophysiological roles [34]. Within this framework, microbiome therapeutics hold multiple functions, with autophagy recognized as one of the most promising and extensively studied mechanisms. Consequently, microbiome-induced autophagy offers significant potential for treating diverse diseases, including infectious and non-infectious conditions. This section provides an overview of autophagy, from its foundational concepts to the molecular mechanisms underpinning this critical process.

### 3.1. The Definition of Autophagy

Autophagy is a highly regulated cellular process essential for degrading and recycling cellular components and plays a pivotal role in the host’s innate immune defense against intracellular pathogens. The term “autophagy” originates from the Greek words “auto”, meaning self, and “phagy”, meaning eating. Essentially, autophagy enables cells to eliminate damaged or obsolete components, such as organelles or protein aggregates, by enveloping them in a double-membraned autophagosome. These autophagosomes then fuse with lysosomes, forming autolysosomes, where lysosomal enzymes degrade the confined material, and the degradation products are not only utilized to generate energy but are also used to recycle macromolecular constituents.

### 3.2. A Brief History of Autophagy Discoveries

The term “autophagy” was first coined by Belgian biochemist Christian de Duve in 1963, following his discovery of lysosomes in 1955. This led to his receipt of the Nobel Prize in Physiology or Medicine in 1974 [35]. In 1962, Thomas Ashford and Keith Porter observed semi-digested mitochondria and endoplasmic reticulum in rat hepatocytes upon treatment with glucagon [36]. Subsequently, de Duve and his colleagues substantiated the hormone’s ability to stimulate autophagy in 1967 [37]. A decade later, Michael Pfeifer elucidated the inhibitory role of insulin, demonstrating its antagonistic effects on autophagy [38]. Subsequently, Per Seglen and Paul Gordon conducted the first biochemical analysis of autophagy, reporting the inhibitory role of 3-methyladenine as a pharmacological agent [39].

From the 1950s to the 1980s, studies on autophagy, primarily based on morphological analyses, were revealed in mammalian systems [40]. Subsequently, advances in understanding and research into autophagy expanded at the molecular level in the yeast *Saccharomyces cerevisiae*, mainly due to Yoshinori Ohsumi’s contributions [41]. Ohsumi identified essential autophagy-related genes (ATGs) by genetically screening autophagy-defective mutants in *Saccharomyces cerevisiae*, thereby elucidating the molecular mechanisms that regulate this process, which earned him the Nobel Prize in Physiology or Medicine in 2016 [42]. Over 37 autophagy-related genes have been identified primarily in *S. cerevisiae*, *Pichia pastoris,* and *Hansenula polymorpha*, highlighting a diverse set of genes crucial for autophagosome formation and the progression of autophagy [43].

Beth Levine’s laboratory first demonstrated the correlation between autophagy and human diseases by identifying Beclin1, a mammalian homolog of yeast ATG6, as a tumor suppressor gene. Subsequent studies elucidated the close association between autophagy and pathological conditions, such as cell growth, cell death, and neurodegeneration [44]. Crucially, researchers have recognized autophagy as a host defense mechanism against bacterial infections, thereby positioning it as an emerging field of research focused on developing novel strategies to combat infectious diseases [45].

### 3.3. Types of Autophagy

Autophagy can be divided into three primary types based on specific functions and mechanisms: micro-autophagy, chaperone-mediated autophagy, and macro-autophagy. Micro-autophagy is a non-selective lysosomal degradation process that directly engulfs cytoplasmic cargo through autophagic tubes sequestered into the lysosomal lumen for degradation [46]. This type of autophagy primarily regulates organelle size, membrane homeostasis, and cell survival under nitrogen restriction [47]. Chaperone-mediated autophagy (CMA) involves selective cargo recognition by cytosolic chaperones and translocation across the lysosomal membrane via a receptor-mediated process rather than engulfing target substrates [48]. This pathway plays a significant role in regulating cellular proliferation, maintaining cellular homeostasis, and responding to various stressors, such as nutritional and oxidative stress [49,50,51].

The third type of autophagy, macro-autophagy, is the most extensively studied and best-characterized autophagic process [52]. In this pathway, cargo is segregated from the rest of the cytosol, forming a double-membrane vesicle known as an autophagosome, which then fuses with lysosomes, leading to the degradation of the engulfed materials [53]. Macro-autophagy facilitates a significant proportion of lysosomal degradation within cells [52]. Generally referred to as “autophagy” in the literature, where the term specifically implies macro-autophagy, it is pivotal in various biological processes and diseases, thus receiving extensive attention in research; therefore, we focus on unraveling the molecular mechanisms underlying this form of autophagy in this review.

### 3.4. Molecular Mechanism of Macro-Autophagy

#### 3.4.1. Formation of the Isolation Membrane

The autophagy machinery’s initial step involves forming an isolation membrane, also known as a phagophore, which begins with nucleation from a specialized region of the endoplasmic reticulum termed the omegasome [54]. To initiate the nucleation of the isolation membrane, activation of the ULK1 complex (ULK1–ATG13–FIP200–ATG101) is required; this complex can be activated in response to autophagy-stimulating signals or nutrient deprivation [55,56]. Another complex, PI3K complex 1, which includes VPS34–VPS15–Beclin1–ATG14, plays a crucial role in forming the isolation membrane [57]. This complex contributes to the production of phosphatidylinositol 3-phosphate (PI3P) at the omegasome, which in turn is essential for recruiting the ATG5–ATG12–ATG16 complex, necessary for the elongation of the isolation membrane [58].

It is noteworthy to mention that there are two primary regulators of autophagy initiation: mTOR and AMPK [59]. The protein mTOR primarily regulates autophagy under various stress conditions: in nutrient-rich conditions, mTOR binds to ULK1, phosphorylates it, and inhibits autophagy, whereas, in nutrient-deprived conditions, mTOR’s phosphorylation sites become dephosphorylated, activating the ULK1 complex and thus stimulating autophagy [60]. Conversely, AMPK induces autophagy in low glucose levels by phosphorylating ULK1 and Beclin1 or suppressing mTOR activities [61].

#### 3.4.2. Isolation Membrane Elongation and Autophagosome Formation

Following the formation of the isolation membrane, the phagophore undergoes elongation through the association with the ATG5–ATG12–ATG16 complex [62]. This complex promotes the conjugation of phosphatidylethanolamine to LC3-I, resulting in the formation of LC3-II, a lipidated form of LC3, which localizes to both sides of the isolation membrane and thus serves as a reliable marker for autophagosome formation [62]. However, as the process progresses, the ATG5–ATG12–ATG16 complex gradually dissociates while the levels of LC3-II increase, ultimately leading to the closure of the isolation membrane and the formation of a mature double-membrane structure known as an autophagosome [63]. Other proteins, such as the SNARE proteins, ATG2, ATG8, ATG9, and WIPIs, also play critical roles in membrane dynamics and autophagosome maturation [64]. Studies have also demonstrated the involvement of Rab33B, a member of the Rab small GTPase family, in the elongation and formation of autophagosomes through interaction with the ATG16L protein [65].

#### 3.4.3. Fusion and Degradation

The terminal step of the autophagy machinery involves the fusion of autophagosomes and lysosomes, facilitating the degradation of sequestered materials. This process is mediated by the association and interaction of specific proteins, where syntaxin 17 on autophagosomes interacts with VAMP8 and SNAP29 on lysosomes, affecting the membrane fusion. Additionally, the Rab7 GTPase, recruited on both autophagosomes and late lysosomes, assists in their docking and fusion [66,67]. The homotypic fusion and protein sorting (HOPS) complex is also instrumental in bringing autophagosomes and lysosomes into proximity to facilitate their fusion [68].

Upon the fusion of autophagosomes and lysosomes, autophagolysosomes are formed, resulting in the degradation of engulfed substances through the action of lysosomal hydrolases, including proteases, lipases, nucleases, and cathepsins [69]. Subsequently, the degraded products are released into the cytoplasm, utilized for energy generation, and recycled to synthesize cellular components [70].

## 4. *Mycobacterium tuberculosis* and Its Pathogenesis

*M. tb* is one of the deadliest pathogens affecting human civilization, responsible for the death of hundreds of millions of lives, and continues to pose a significant health, social, and economic burden worldwide [71]. Study reports suggest that *M. tb* emerged as a human pathogen in Africa around 70,000 years ago and spread out of the continent following human migrations [71]. The ancient *M. tb* strains underwent significant evolution and acquired the capability to reactivate disease after long periods of latent infection [72]. Additional research revealed the evidence of fatalities from *M. tb* dating back as far as 3000 years in Egyptian mummies, underscoring its prolonged presence in human history. More recently, the signs of pulmonary tuberculosis were identified in a New World mummy from Peru, dating back to 700 AD [73]. Tuberculosis embodies complex interactions between the host’s immune responses and the pathogen’s evasion strategies [74]. Therefore, herein, we aim to briefly focus on the pathogenesis of *M. tb* and the virulence factors that contribute to the survival of this lethal pathogen within the host.

Upon inhalation, *M. tb* enters the lungs and primarily interacts with alveolar macrophages as well as other phagocytic cells, initiating the host’s innate immune response [75]. Simultaneously, the bacterium evades the host defense mechanisms by inhibiting phagolysosome maturation, thus avoiding degradation in phagolysosomes [76]. Furthermore, the genome of the *M. tb* pathogen encodes type VII secretion systems (TSSS), which support its survival and proliferation [77]. Among the TSSS, Esx-1 has been extensively studied and plays a central role in pathogenesis by disrupting the phagosomal membrane with other effectors, such as Esx-A/ESAT-6 and Esx-B/CFP-10, enabling bacterial replication in the cytosol [74,78]. In addition, Esx-A and Esx-B (effectors of Esx-1) serve as antigens recognized by CD4+ and CD8+ T cells, thus activating the adaptive immune system [79]. Another secretory protein, identified as the enhanced intracellular survival (EIS) protein, has been demonstrated to play a vital role in the survival of *M. tb* within human macrophages [80]. The EIS protein disrupts the Th1 protective response, thereby enhancing the survival of intracellular *M. tb* pathogens [81]. Studies have also demonstrated that it suppresses host defense mechanisms by negatively modulating inflammation, autophagy, and cell death in a redox-dependent manner [74]. Several mycobacterial lipids and proteins contribute to the pathogenesis of *M. tb*. Among these, PtpA (a tyrosine phosphatase) and NdkA (a GTPase-activating protein) hinder the activation of Rab7, which is essential for phagosome maturation [82]. Furthermore, ManLAM, a mycobacterial cell wall protein, disrupts a calmodulin signaling pathway, decreasing the levels of phosphatidylinositol 3-phosphate (PI3P), a critical component for phagosome maturation. This reduction was observed when mycobacterial phagosomes were compared to non-mycobacterial or non-viable mycobacterial phagosomes, indicating the impairment of mycobacterial phagosomes [83]. Additionally, *M. tb* proteins, such as PknG, LpdC, Zmp1, and TlyA, are involved in the arrest of phagosome maturation, thereby playing a role in enhancing the survival of these pathogens within host immune cells [84].

## 5. Immune Response to *Mycobacterium tuberculosis*

Microbiome therapeutics represent an innovative concept in tuberculosis treatment, primarily by modulating the host’s immune system. Additionally, they play a crucial role in protecting tissues from damage caused by excessive inflammation triggered by pathogens, achieved by regulating proinflammatory cytokines and chemokines. This dual functionality of microbiome therapeutics positions them as a potential strategy for managing tuberculosis innovatively. Before examining why microbiome therapeutics might serve as a trigger to eradicate tuberculosis effectively, it is essential to first investigate the immune system’s response to *M. tb.*

*M. tb* is an intracellular pathogen that can persist within host immune cells as a latent *M. tb* infection for a prolonged period without manifesting visible symptoms or active disease. Individuals with a latent *M. tb* infection have a 5–10% lifetime risk of developing the active disease, with immunocompromised conditions further increasing this risk. This scenario highlights the potential of microbiome therapeutics to manage *M. tb* infection by enhancing the host’s innate defense mechanisms. However, as the lungs are the primary infection site for *M. tb,* alveolar macrophages in the lungs serve as the first line of defense upon the pathogen’s entry. The complement receptor (CR3) serves as the macrophages’ primary receptor, facilitating the uptake of *M. tb*. Besides CR3, other receptors such as CR1, CR4, CD14, mannose receptors, and scavenger receptors also recognize and bind to *M. tb* [85,86]. Moreover, the uptake of *M. tb* is stimulated by pulmonary surfactant protein A in human macrophages [87,88]. However, once internalized, *M. tb* adopts strategies to evade the host’s innate microbicidal activities by inhibiting the maturation of the *M. tb* phagosome. This inhibits lysosomal biogenesis and, thus, the acquisition of lysosomal components [89,90]. Ultimately, this formidable pathogen can effectively evade the host immune system. Studies show that the *M. tb* phagosome interacts with Rab5, thereby reducing the recruitment of the early endosomal autoantigen 1 (EEA1), an effector molecule of Rab5 [91,92], playing a key role in the autophagic process. Another study reported that *M. tb* phagosomes lack phosphatidylinositol 3-phosphate, a primary activation product of type III phosphatidylinositol 3-kinase and hVPS34, crucial for the retention of EEA1 on the endosomal membrane [93]. Further research identified several factors that inhibit the maturation of *M. tb* phagosomes, including the suppression of actin assembly, the prevention of iron acquisition, and mannosylated lipoarabinomannan (ManLAM) [76,94,95]. While surviving inside macrophages, *M. tb* induces the production of proinflammatory cytokines such as interleukin-1 (IL-1), interleukin-12 (IL-12), interleukin-17 (IL-17), and tumor necrosis factor-α (TNF-α). These cytokines stimulate other immune cells, forming an organized structure known as a granuloma, a hallmark of tuberculosis infection [96,97]. Although granulomas are considered to restrict the dissemination of *M. tb* infection, some bacilli can still survive and thrive within these structures for extended periods by modulating the host’s immune response. Eventually, they can escape these defenses, leading to clinical disease [98,99].

As the innate immune response is not fully capable of eliminating intracellular *M. tb*, the dissemination of *M. tb* further stimulates the acquired immune system of the host. Among the immune cells of adaptive immunity, T cells play a vital role in reducing the *M. tb* burden, thereby affording protection to the host [100,101]. The T cell response commences following the presentation of *M. tb* or *M. tb* antigens to the lymph node by dendritic cells expressing MHC class II (also known as classical antigen-presenting cells (APC)), where the acquired immune response is coordinated. However, some studies report the initiating of the acquired immune response in mice lacking lymph nodes [102,103]. Consequently, dendritic cells initiate and stimulate the expansion of antigen-specific naïve CD4^+^ and CD8^+^ T cells, positioning them as the primary immune cells of the adaptive immune system to encounter the *M. tb* pathogens [104,105]. These activated cells exhibit polyfunctional activities and can produce IFN-γ, TNF, IL-2, and IL-12. Studies have shown that mice deficient in the Th1-polarizing cytokines IL-12/IFN-γ succumbed early after exposure to *M. tb* infection. Additionally, the Th1-type immune response can stimulate the infected macrophages to acquire antimicrobial functions, thus aiding in eliminating *M. tb* pathogens. Moreover, IFN-γ can enhance the accumulation of CD4^+^ and cytotoxic T lymphocytes at the infection site, thereby improving antigen presentation and facilitating *M. tb* killing activities.

To combat *M. tb* pathogens, the host’s immune system is activated, leading to an increased level of inflammation as well as the overexpression of cytokines, chemokines, matrix metalloproteinases-1 (MMP-1), and matrix metalloproteinases-3 (MMP-3) [106,107]. In active adult pulmonary TB cases, significant increases in MMP-1 and MMP-3 have been noted in sputum and bronchoalveolar lavage fluid, marking them as indicators of cavitation [108]. Research has also shown that the pronounced upregulation of type I IFN during *M. tb* infection leads to localized tissue damage [109]. Other studies have indicated that increased TNF-α, IL-1β, IL-6, IL-8, and the cytotoxic T cell response can cause lung tissue damage during tuberculosis [110,111].

This discussion emphasizes that the host defense system can combat *M. tb*, although *M. tb* has developed strategies to evade these defenses. It further demonstrates that while protecting cells from *M. tb*, the host immune response can become overstimulated, which may be detrimental to the host’s tissues. Considering these factors, microbiome therapeutics can be a practical approach for managing tuberculosis. This modality demonstrates efficacy in reducing the burden of *M. tb* and aids in regulating inflammation, thus offering a balanced and effective treatment strategy.

## 6. The Potential of Microbiome Therapeutics Targeting Autophagy in Controlling Tuberculosis

The role of microbiome therapeutics in maintaining well-being has been discussed in previous sections. Microbiome therapeutics encompasses many strategies, including probiotics, prebiotics, postbiotics, fecal microbiota transplantation, engineered bacteria, and combinational therapies. These modalities focus on modulating the host immune system and targeting the microbial ecosystem to promote a healthy state. Among these strategies, probiotics are recognized as one of the most direct and established forms of microbiome therapeutics. They offer a novel and effective method for managing diseases, particularly in reducing the burden of *M. tb,* as evidenced by recent studies. However, a brief overview of probiotics will be provided before delving into the primary discussion.

### 6.1. What Is a Probiotic?

The term “probiotics” is derived from Latin (pro) and Greek (bios), meaning ‘for life’ in Greek, and its definition has undergone a remarkable evolution [112]. In 1954, the German scientist Ferdinand Vergin introduced the term *probiokita* to describe “active substances essential for a healthy development of life” [113]. The term probiotic, first coined by Lilley and Stillwell in 1965, originally described substances that stimulate the growth of microorganisms. Over the years, its meaning expanded: Sperti broadened its scope in 1971, and Parker linked it to maintaining intestinal microflora balance in 1974 by proposing that probiotics should include microbial organisms and other substances. Fuller further refined the definition in 1989, articulating it as “a live microbial feed supplement which beneficially affects the host animal by improving its intestinal microbial balance” [114]. In 2013, an expert panel convened by the International Scientific Association for Probiotics and Prebiotics (ISAPP) reiterated the FAO/WHO definition, stating that probiotics are “live microorganisms which, when administered in adequate amounts, confer a health benefit on the host” [115].

### 6.2. Development Stages of Probiotics in Health Promotion from the Ancient to the Modern Era

The history of probiotics has evolved concurrently with human civilization, traceable to ancient times [116]. Long before recognizing the existence of probiotic microorganisms, fermented products such as beer, bread, wine, kefir, kumis, and cheese were extensively used for both nutritional and therapeutic purposes [116]. The Vedic hymns of India, composed before 2000 BC, indicate that the Hindu population had incorporated fermented milk products into their diets since antiquity [117]. Additionally, between 2000 and 3000 BC, various civilizations, including the Egyptians, Greeks, and Romans, extensively documented their widespread use of fermented milk, cheese, and butter [118]. Virtually every civilization has adopted some form of food fermentation in its culinary traditions. Notably, the cultures of Japan, China, and Korea have relied on fermentation to pickle various vegetables such as cabbage, turnip, eggplant, cucumber, onion, squash, and carrots throughout the centuries. Historical records also note the customary supplementation of fermented vegetables to workers for their health during the construction of the Great Wall of China around 300 BC [119].

These historical perspectives underscore the symbiotic relationship between bacteria and humans, marking a transition from perceiving bacteria as merely pathogenic. Regarding this, Elie Metchnikoff’s studies in 1905 linked the increased longevity of the Bulgarian population to the consumption of *Lactobacilli* from yogurt [120]. In the 1930s, Japanese microbiologist Minoru Shirota noted bacterial flora’s survival during gut transit, which led to the discovery of the *Lactobacillus casei* strain *shirota* and the subsequent development of the commercially successful Yakult drink [121]. In 1973, urologist Andrew Bruce suggested using *Lactobacilli* as a probiotic strain for the urogenital tract, highlighting the complexity of the intestinal microbiota [122]. The Human Microbiome Project (HMP), launched in 2008, employed high-throughput technologies to analyze samples from 300 healthy volunteers. This led to the isolation and sequencing of nearly 1300 reference strains, demonstrating the beneficial impact of microorganisms on their hosts [123].

Initially linked to fermentation and promoting health, probiotics now predominantly address various diseases and disorders, primarily as Live Biotherapeutic Products (LBPs). They have demonstrated beneficial effects on several non-infectious ailments, including obesity, insulin resistance, wound healing, diabetes, allergies, irritable bowel syndrome, cancer, eczema, atopy, inflammation, hepatic encephalopathy, gastrointestinal disorders, pathological neonatal jaundice, and psychological stress [124,125].

Moreover, they demonstrated efficacy against a wide range of diseases caused by pathogenic microorganisms, including *Helicobacter pylori*, *Salmonella*, enterotoxigenic *E. coli*, *Candida spp.*, *Streptococcus pneumoniae*, *Campylobacter*, carbapenem-resistant *Enterobacteriaceae* (CRE), multidrug-resistant *Acinetobacter baumannii*, multidrug-resistant *Pseudomonas aeruginosa*, vancomycin-resistant *Enterococcus* (VRE), and methicillin-resistant *Staphylococcus aureus* (MRSA), as well as both drug-sensitive and resistant *Mycobacterium tuberculosis* strains [126,127,128,129,130,131,132,133,134,135,136,137]. Notably, the FDA has already approved probiotic-based drugs such as SER-109 and Rebyota [138] that are currently available on the market, while several others are undergoing clinical trials and are expected to be introduced shortly.

### 6.3. Emerging Concept of Probiotics as Autophagy Activators and Their Impact on Host Health

Probiotics confer benefits to the host through multiple proposed pathways, including autophagy, a significant and increasingly studied mechanism. In this context, we aim to explore research that has utilized probiotics to stimulate autophagy and their associated health benefits.

The mechanisms by which probiotics confer health benefits through the induction of autophagy have been extensively studied in in vitro and in vivo models. M.A. Engevik et al., 2019 demonstrated that the probiotic *Bifidobacterium dentium* can upregulate autophagic signaling pathways, enhancing the mucus layer and goblet cell function, using an in vitro model [139]. Another study demonstrated the capacity of the probiotic strains *Lactobacillus* and *Bifidobacterium* to induce autophagy in bone marrow-derived dendritic cells (BMDC), suggesting autophagy as a potential mechanism within the regulatory machinery of probiotics [140]. Svetlana et al., 2020 reported that *Lactobacillus brevis* BGZLS10-17 could stimulate autophagy in mesenteric lymph node cells (MLNC), including CD4+ and CD8+ T lymphocytes, NK and NKT cells, as well as antigen-presenting cells, thus providing significant immunoregulatory effects on MLNC [141]. The probiotic strain *Bacillus* SC06 has been shown to attenuate oxidative stress-induced intestinal injury by stimulating autophagy in rat jejunum and IEC-6 cells [142]. *Bifidobacterium breve* has been found to upregulate autophagy via the MAP kinase signaling pathway, providing cytoprotection to intestinal epithelial cells against inflammation-induced stress [143]. Another study demonstrated that *Bifidobacteria* confers enteroprotective effect against LPS-induced toxicity and regulates gut homeostasis by stimulating autophagy in an in vitro model [144].

In vivo studies have also demonstrated the robust immunomodulatory effects of probiotic-mediated autophagy on host immunity, thereby conferring health benefits. A study reported that a probiotic formulation (composed of nine live probiotic strains) partially restored the impaired autophagic pathway (impaired autophagy is associated with a variety of disorders/diseases), leading to a reduction in the progression of Alzheimer’s disease in a mouse model [15]. Yang et al., 2020, demonstrated that the administration of *Bacillus amyloliquefaciens* in the diet increased the growth performance of piglets by enhancing the intestinal autophagic pathway [145]. The probiotic *Bacillus* SC06 strain has been shown to alleviate disorders induced by oxidative stress through the stimulation of the autophagic process in an animal model [142]. Probiotic-mediated autophagy also plays a critical role in embryo development. For instance, studies have shown that the parental administration of *Lactobacillus rhamnosus* maintains the balance between apoptosis and autophagy, improving follicular survival [146], and is associated with embryo development in zebrafish [147].

Probiotic-mediated autophagy has also demonstrated its efficacy against a spectrum of infectious diseases. For example, research has shown that the probiotic *Bacillus amyloliquefaciens* SC06 can stimulate autophagy in murine RAW264.7 macrophage cells, thus providing protection against *Escherichia coli* and preventing bacterial infections in the intestine [16]. *Rong* et al. demonstrated that lactic acid bacterial strains increase the autophagic response both in vitro and in vivo and provide protection against *Salmonella* infection, suggesting their therapeutic and preventative efficacy in inflammatory bowel disease [148]. Another study highlighted the protective effect of *Lactobacillus salivarius* AR809 in a *Staphylococcus aureus*-induced pharyngitis model through activation of the autophagic process [149].

This discussion shows that probiotics possess robust immunomodulatory capacities that stimulate autophagy, providing numerous host benefits, particularly protection against pathogenic microorganisms. Thus, they could serve as alternative sources to the current antibiotics. In this regard, probiotics also hold potential as microbiome therapeutics against *M. tb*, as recent studies have demonstrated their effectiveness in reducing this pathogen’s burden. Therefore, in the subsequent section, we will explore probiotic-mediated autophagy and its potential as a microbiome therapeutics approach to controlling TB.

### 6.4. The Potential of Probiotic-Mediated Autophagy in Managing Tuberculosis

The use of probiotics targeting autophagy as a defense mechanism represents an emerging concept in microbiome therapeutics for managing TB, though it is still in its nascent stages. Recent research revealed the effectiveness of this approach in clearing *M. tb* from infected cells. This study demonstrated a promising intracellular killing effect of the probiotic strain *Lacticaseibacillus rhamnosus* against both drug-sensitive and drug-resistant *M. tb* strains. Autophagy was identified as a potential intracellular killing mechanism, accompanied by the enhanced expression of autophagy-related genes and vesicle colocalization [137]. The research team further extended the study, focusing on the anti-*M. tb* molecular mechanisms related to autophagy [150]. They discovered that the probiotic strain significantly increased the expression of LC3-II markers while reducing the expression of p62, as evidenced by immunoblotting and immunofluorescence methods. LC3-II (microtubule-associated protein 1 light chain 3) is a soluble protein (16~18 kDa) ubiquitously found in mammalian tissues and cultured cells [151]. As the autophagic process progresses, a cytosolic form of LC3 (LC3-I) is conjugated to phosphatidylethanolamine to form the LC3-phosphatidylethanolamine conjugate (LC3-II), which is then recruited to autophagosomal membranes [151]. On the other hand, p62, a well-known autophagic substrate, is typically degraded during the fusion of the autophagosome with the lysosome, resulting in the formation of an autolysosome, a late stage of autophagy [152]. However, the study also found that the probiotic strain enhanced the generation of acidic vesicular organelles and LAMP1 proteins, indicators of autolysosome formation [153,154]. Consequently, the enhanced autophagy led to the elimination of the *M. tb* burden from the infected cells, demonstrating the potential of probiotics-induced autophagy as a defense mechanism in managing TB. Another study conducted by Hossain et al., 2024, showed the effectiveness of probiotic strains in reducing the *M. tb* burden with a heightened expression of autophagy-related genes, which aligns with previous studies and further indicates the potential of probiotics against *M. tb* pathogens [155]. Another research study reported the effectiveness of the probiotic strain *Bacillus subtilis* against extensive drug-resistant pulmonary *M. tb* in an in vivo model [156]. Mice in the microbiome therapeutics-treated group survived throughout the experimental period, while mice in the untreated group died before the study concluded. Probiotic treatment reduced the degree of *M. tb* in the lungs with fewer granulomas and restored the lung microbiota, which was disrupted in the untreated group. Interestingly, the study also noted increases in autophagy when the probiotic strain was introduced to macrophage cells. This was evidenced by increased LC3–vesicle colocalization, a hallmark of autophagic progression, and the upregulation of autophagy-related genes, indicating autophagy as a host defense mechanism that might be involved in increasing the survival rate of the mice.

Maintaining an appropriate inflammatory response is crucial in TB management, as it is closely associated with host defense mechanisms. Studies have shown the role of probiotics in modulating the inflammatory response, thus aiding in the control of TB. In this regard, a study conducted by *Darab* et al. demonstrated that lactic acid bacteria can enhance the immune response of mononuclear phagocytes to *M. tb* antigens by increasing the production of autophagy-promoting factors such as IFN-γ and NO [157]. The study also revealed that probiotics can reduce the Th2 autophagy-inhibiting cytokines, including IL-4 and IL-13. These cytokines can impair the autophagic process and hinder the clearance of intracellular *M. tb* in both murine and human macrophages [158]. *M. tb* itself upregulates the Th2 immune response while downregulating the Th1 response, thereby undermining natural protective immunity, including autophagy, and maintaining a lower ratio of IFN-γ to IL-4 [159,160]. Therefore, maintaining an appropriate level of IFN-γ is vital for controlling TB. In this context, studies have demonstrated that supplementing probiotics in TB patients during the intensive phase of treatment can significantly increase the levels of IFN-γ and IL-12 [161]. Pretreatment of *M. tb*-infected macrophages with IFN-γ increases the fusion of *M. tb*-containing phagosomes with lysosomes, thereby enhancing the intracellular killing of this pathogen [162]. A study demonstrated that silencing Beclin1, a key component of the autophagy signaling pathway, in murine macrophages completely nullifies the effects of IFN-γ on the maturation of BCG-containing phagosomes, thereby affirming the association between the autophagic process and IFN- γ [163]. Consequently, maintaining a balanced immune response, especially the ratio of Th1/Th2 cytokines, is crucial in managing TB, a focus that has recently attracted considerable attention. In this context, probiotics are increasingly acknowledged for their role in modulating the appropriate immune response and enhancing autophagy, which can subsequently control *M. tb* pathogens.

Autophagy has been identified to play a role in dendritic cell biology. As key professional antigen-presenting cells, dendritic cells are crucial for innate and adaptive immune responses. They exhibit a unique capacity to activate naïve T cells, which play a pivotal role in protective immunity against infections and regulating immune homeostasis and tolerance. For these processes to function optimally, robust endocytic and lysosomal activities closely associated with autophagy are essential. The involvement of autophagy-related proteins, especially LC3, in dendritic cell biology has been extensively studied and highlighted in numerous reports [164,165]. Based on these findings, it can be hypothesized that a specific probiotic strain could also stimulate dendritic cells, as there exists a strong correlation between autophagy and the activation of dendritic cells in the existing literature. Moreover, in addition to activating autophagy, probiotics themselves may enhance the function of dendritic cells; for instance, one study demonstrated that supplementation with *Lactobacillus plantarum* MTCC 2621 restored the expression of macrophage-inducible C-type lectin on lung dendritic cells and enhanced the anti-TB response. From these perspectives, probiotics or probiotic-mediated autophagy not only reduce the TB burden but also have the potential to augment the activation of dendritic cells, thus contributing to a further decrease in *M. tb* pathogens.

*M. tb* pathogens block phagosome fusion with lysosomes, thus evading the host defense system. Studies indicate that the physiological or pharmacological induction of autophagy can overcome the trafficking block imposed by this pathogen, resulting in the formation of phagolysosomes and leading to their degradation [162]. This study highlights the role of probiotics in controlling TB, given that the ability of probiotics to induce autophagy is well-established and discussed in the previous section. Furthermore, a recent study confirmed the protective role of autophagy against high doses of *M. tb* infections at the in vivo level [13]. The findings demonstrate that disruption of the autophagy gene function in CD11c^+^ cells (lung macrophages and dendritic cells) results in the increased accumulation of polymorphonuclear myeloid-derived suppressor cells that harbor high levels of *M. tb* pathogens. The study also revealed that the accumulated polymorphonuclear myeloid-derived suppressor cells are closely associated with reduced T cell proliferation in the lung, indicating a defect in the T cell response and thereby preventing the control of *M. tb* pathogens. Applying probiotics as microbiome therapeutics could be a potential strategy to control this lethal pathogen by stimulating the autophagic process. Additionally, a study by *Eliseo* et al. demonstrated that transgenic mice deficient in ATG5 in the myeloid lineage, including macrophages [12], are more susceptible to *M. tb* infection, leading to an increased bacilli burden and earlier mortality compared to control groups. They also reported excessive pulmonary inflammation characterized by neutrophil infiltration in the target group mice, demonstrating the role of autophagy in mitigating excessive inflammatory responses in the host. In this regard, probiotics may serve as an emerging approach capable of reducing the *M. tb* burden by enhancing autophagic processes and regulating excessive inflammation. Meanwhile, a study indicated autophagy’s direct *M. tb* killing effect beyond its traditional bactericidal compartments, such as phagolysosomes [166]. This study demonstrated that p62, an accessory autophagic substrate protein, can transport cytosolic proteins to phagolysosomes, which are converted into neo-antimicrobial peptides from their harmless forms following digestion, thereby aiding the reduction in the *M. tb* burden. Probiotics can enhance this immunological function of p62 by stimulating autophagy, which leads to the production of anti-TB peptides.

**Figure 1:**
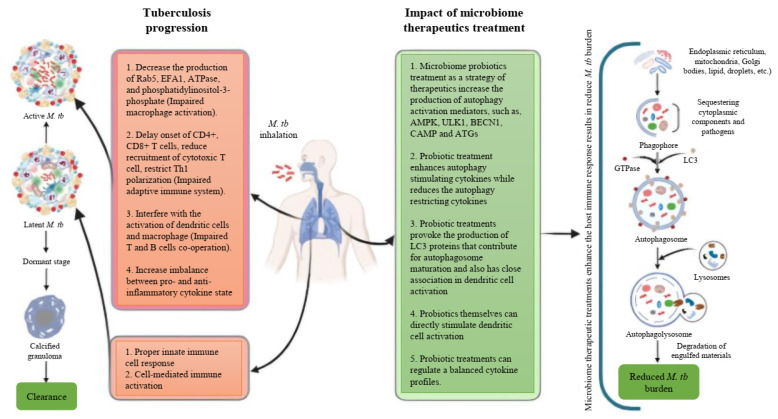
Schematic summary of the tuberculosis disease progression and the impact of microbiome therapeutics on the host’s immune system.

**Figure 2:**
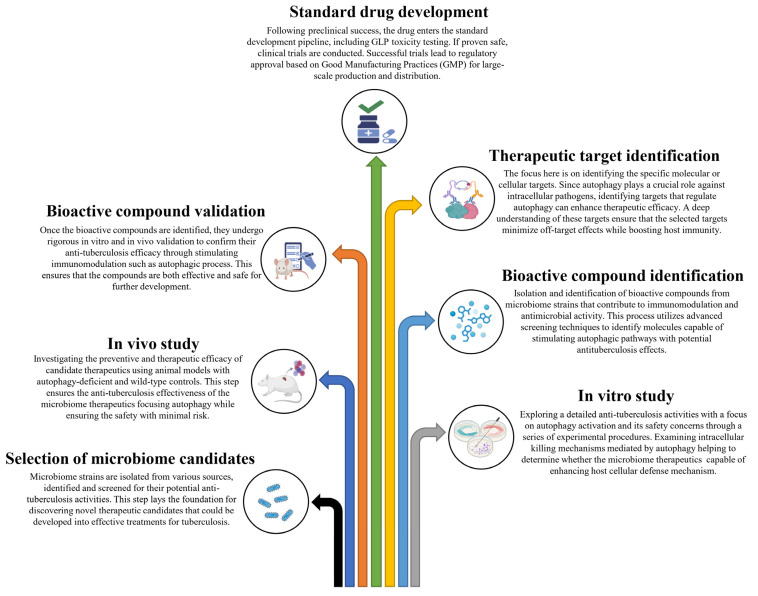
A stepwise approach to developing microbiome therapeutics for combating *Mycobacterium tuberculosis* through host immunomodulation.

Based on these studies, it is evident that autophagy serves a protective role against *M. tb* pathogens, and it is reasonable to conclude that inducing autophagy in host cells using non-toxic agents could potentially be a therapeutic strategy for controlling this lethal pathogen. In these circumstances, probiotics emerge as promising microbiome therapeutics in the management of TB, considering their proven ability to induce autophagy and their efficacy in modulating excessive inflammation. In this context, we have proposed a schematic summary of tuberculosis progression along with the potential impact of microbiome therapeutics focusing on the effects of autophagy on the host’s immune system (Figure 1). We have also proposed a stepwise approach to developing microbiome therapeutics for combating *M. tb* through host immunomodulation (Figure 2).

## 7. Limitations of Current Drug Regimens for Treating Drug-Resistant Tuberculosis

The emergence of multi-drug-resistant (MDR-TB), extensively drug-resistant (XDR-TB), and totally drug-resistant (TDR-TB) *M. tb* strains have rendered both first-line (Isoniazid, Rifampicin, Pyrazinamide, Ethambutol, and Streptomycin) and second-line drug regimens (Amikacin, Kanamycin, Capreomycin, Levofloxacin, Moxifloxacin, Ofloxacin, and Ethionamide) ineffective, posing a significant threat to global human health. According to the 2023 WHO report, the number of individuals affected with drug-resistant *M. tb* strains has increased compared to previous years. The emergence of resistance, even to newer drugs such as bedaquiline, delamanid, and linezolid, designed to combat MDR-TB, has been observed [167,168].

Additionally, standard TB treatment regimens require extended treatment durations with combination therapies. According to the WHO, the standard treatment protocol for new cases entails two months of isoniazid, rifampicin, pyrazinamide, and ethambutol, followed by four months of isoniazid and rifampicin. For retreatment, the recommended WHO regimen comprises 2 months of streptomycin, isoniazid, rifampicin, pyrazinamide, and ethambutol, followed by 1 month of isoniazid, rifampicin, pyrazinamide, and ethambutol, and concluding with 5 months of isoniazid, rifampicin, and ethambutol, and a combination of rifampicin, pyrazinamide, and ethambutol for 6–9 months. In contrast, MDR-TB regimens require a more extended treatment period, spanning 18–24 months. Prolonged durations enhance the risk of treatment discontinuation and relapse. Furthermore, second-line drugs often induce severe side effects, [169] diminishing patients’ quality of life and adherence to therapy. Patients co-infected with TB and HIV experience complex drug interactions between anti-TB drugs and antiretroviral therapy (ART), further complicating treatment [170]. The economic burden of TB medications and extended hospital stays is often overwhelming for patients in low-income settings, restricting access to effective treatments. Additionally, *M. tuberculosis* can manipulate the host immune system, including autophagy pathways, to persist within macrophages, thereby circumventing immune-mediated clearance. Current drug regimens do not adequately target these bacterial survival strategies, frequently resulting in persistent infection and incomplete eradication [171].

## 8. Advantages of Microbiome Therapeutics over Conventional Drugs

Microbiome-based therapeutics have gained central attraction within the scientific community for managing various diseases in the era of antibiotics. Here, we aim to reflect on some of these benefits compared to conventional drug regimens. Conventional treatments have numerous issues, including antibiotic resistance, decreased drug sensitivity, and low drug specificity [172], thus hindering the successful management of tuberculosis. Conversely, microbiome therapeutics introduce a novel approach capable of overcoming these limitations. Unlike conventional anti-TB therapies, which often target pathogens indiscriminately and severely disrupt the normal microbiota, microbiome therapeutics address the root cause of the infection rather than merely addressing the symptoms. They possess a unique ability to restore microbial diversity and support immune homeostasis, promoting a more effective way to manage TB. Lung microbiota influence TB pathogenesis [173], but microbiome-based interventions promote beneficial bacteria while suppressing pathogens, providing a less favorable environment for *M. tb,* thus reducing the risk of resistance [174].

Additionally, microbiome-based therapeutics can regulate cytokine profiles, reducing excessive inflammation while promoting effective immune responses [175]. This dual action mitigates the tissue damage commonly associated with TB. Furthermore, they exhibit fewer side effects than conventional therapies, making them safer, more reliable, and viable long-term TB management options. Moreover, the growing availability of cost-effective metagenomics profiling enables more precise applications of these therapies [176], such as tailoring personalized medicine based on an individual’s microbiome profile, thus offering more effective interventions to manage TB [177]. Beyond TB, microbiome interventions may offer additional benefits, such as improved metabolic health, reduced susceptibility to co-infections, and enhanced recovery from co-morbidities.

## 9. Challenges and Future Perspectives of Microbiome Therapeutics

The potential of microbiome-based therapies to modulate autophagy offers a promising strategy for combating drug-resistant tuberculosis. However, the development of microbiome-based therapeutics faces several challenges. A significant obstacle is the variability in host responses to these treatments. Differences in individuals’ microbiomes, immune system function, and genetic predisposition can lead to inconsistent therapeutic outcomes, thereby complicating the development of universally effective treatments [178]. Furthermore, ensuring that microbiome-based therapeutic interventions effectively target macrophages or other cells infected with *M. tb* presents a complex task [179]. Most studies are still in the preclinical phase, focusing on elucidating the molecular mechanisms through which *M. tb* disrupts autophagy. Nevertheless, translating this knowledge into clinical therapies remains a significant challenge. Safety concerns and regulatory obstacles further complicate the development of microbiome-based autophagy therapies. The excessive modulation of autophagy could result in unintended consequences [180]. Additionally, maintaining the stability and viability of microbiome-based therapeutic products throughout manufacturing, storage, and distribution poses challenges. Moreover, regulatory authorities such as the U.S. Food and Drug Administration (FDA) and the European Medicines Agency (EMA), which have traditionally focused on chemically defined drugs [181], face complications in evaluating the safety, efficacy, and quality control of microbiome-based therapies, which do not fit these established categories.

These challenges necessitate more thorough and targeted research to advance this evolving therapeutic strategy. In this context, the gut microbiota may play a crucial role as they impact lung immunity via the gut–lung axis, activating immune cells such as macrophages, thereby modulating autophagy pathways critical for combating *M. tb* [182]. Studies indicate that secondary metabolites from gut microbes exhibit anti-mycobacterial activity, suggesting their potential utility as adjunct therapies [183]. Advanced technologies such as meta-transcriptomics, proteomics, and metabolomics can be employed extensively, revealing new therapeutic targets and enhancing our understanding of microbiome–host interactions in TB pathology.

Moreover, emerging technologies such as synthetic biology and CRISPR-Cas9 editing hold transformative potential for developing microbiome-based autophagy therapies. Synthetic biology enables the engineering of probiotic strains that produce metabolites to enhance autophagy or to counteract the evasion strategies of *M. tb* [17,184]. CRISPR technology provides precise tools for editing bacterial genomes, which facilitates the creation of microbiome-based therapeutics with specific functions [17]. For instance, modifying gut bacteria to overproduce anti-mycobacterial compounds or modulating host immune responses could greatly enhance TB treatment outcomes. Profiling microbial communities and their functional metabolites could lead to tailored treatments, accelerating the discovery of novel therapeutic targets and predictive biomarkers for personalized interventions.

Microbiome drug development that focuses on autophagy as a therapeutic strategy for tuberculosis has immense potential to transform the management of DR-TB. However, realizing this potential requires addressing the current challenges through rigorous mechanistic studies, utilizing advanced technologies, and adopting personalized approaches. Integrating microbiome-based therapies with conventional anti-TB drugs and innovations in synthetic biology and precision medicine could create more effective and sustainable solutions. As research progresses, multidisciplinary collaboration and extensive clinical trials will be keys to translating these promising insights into tangible clinical benefits.

## 10. Conclusions

In conclusion, tuberculosis continues to represent a significant threat to global health, mainly due to the emergence of drug-resistant *Mycobacterium tuberculosis* strains and the resurgence of this disease. Existing drug regimens exhibit limitations that underscore the urgent need for the development of novel strategies to manage tuberculosis. Within this framework, microbiome therapeutics targeting autophagy, an innate defense mechanism, have emerged as a viable approach to address these challenges. Microbiome therapeutics demonstrate potential in reducing pathogen load and modulating inflammation, thus positioning them as a promising strategy for TB management. However, research into microbiome therapeutics for tuberculosis is still in its nascent phase, highlighting the critical need for more thorough studies to develop effective anti-tuberculosis therapies.

## Data Availability

No new data were created or analyzed in this study.

## Abbreviations

Tuberculosis (TB), *Mycobacterium tuberculosis* (*M. tb*), Food and Drug Administration (FDA), World Health Organization (WHO), multidrug-resistant (MDR), rifampicin-resistant TB (RR-TB), extensively drug-resistant TB (XDR-TB), next-generation sequencing (NGS), latent tuberculosis infection (LTBI), autophagy-related genes (ATGs), chaperone-mediated autophagy (CMA), phosphatidylinositol 3-phosphate (PI3P), homotypic fusion and protein sorting (HOPS), type VII secretion systems (TSSS), enhanced intracellular survival (EIS), complement receptor (CR), endosomal autoantigen 1 (EEA1), mannosylated lipoarabinomannan (ManLAM), interleukin-1 (IL-1), interleukin-12 (IL-12), interleukin-17 (IL-17), tumor necrosis factor-α (TNF-α), antigen-presenting cells (APC), metalloproteinases-1 (MMP-1), matrix metalloproteinases-3 (MMP-3), International Scientific Association for Probiotics and Prebiotics (ISAPP), Live Biotherapeutic Products (LBPs), lymph node cells (MLNC), microtubule-associated protein 1 light chain 3 (LC3-II), totally drug-resistant tuberculosis (TDR-TB), antiretroviral therapy (ART), European Medicines Agency (EMA).
